# Assessment of Molecular Markers in Pediatric Ovarian Tumors: Romanian Single-Center Experience

**DOI:** 10.3390/ijms25126752

**Published:** 2024-06-19

**Authors:** Ovidiu Bîcă, Carmen Iulia Ciongradi, Diana Benchia, Ioan Sârbu, Mirabela Alecsa, Alexandra Elena Cristofor, Delia Elena Bîcă, Ludmila Lozneanu

**Affiliations:** 1 2nd Department of Surgery—Pediatric Surgery and Orthopedics, “Grigore T. Popa” University of Medicine and Pharmacy, 700115 Iași, Romania; 2 Department of Mother and Child, “Grigore T. Popa” University of Medicine and Pharmacy, 700115 Iași, Romania; 3 Department of Obstetrics and Gynecology, “Grigore T. Popa” University of Medicine and Pharmacy, 700115 Iași, Romania; 4 Department of Clinical Pharmacology, “Grigore T. Popa” University of Medicine and Pharmacy, 700115 Iași, Romania; 5 Department of Morpho-Functional Sciences I—Histology, “Grigore T. Popa” University of Medicine and Pharmacy, 700115 Iași, Romania

**Keywords:** ovarian, tumors, pediatric, markers, SALL4, OCT3/4, immunohistochemistry

## Abstract

Pediatric ovarian tumors exhibit unique diagnostic and therapeutic challenges. This study evaluates the expression of SALL4 and OCT3/4 biomarkers in pediatric ovarian tumors and their associations with tumor subtype, stage, and clinical outcome. A retrospective analysis was conducted on 64 patients under 18 years old, examining demographic data, tumor characteristics, immunohistochemical staining, and clinical outcomes. Our results show that SALL4 was significantly expressed in adenocarcinoma, dysgerminoma (DSG), mixed germ cell tumors (GCTs), and immature teratoma, while OCT3/4 was highly expressed in DSG and mixed GCTs. Both markers are associated with a higher tumor grade and stage, indicating a more aggressive disease. The SALL4 positivity expression was correlated with high alpha fetoprotein (AFP) and lactate dehydrogenase (LDH) levels, while OCT3/4 positivity significantly predicted the risk of subsequent metastasis. The mean progression-free survival (PFS) was notably shorter in patients with positive markers. These findings underscore the diagnostic and prognostic value of SALL4 and OCT3/4 in pediatric ovarian tumors, aligning with previous research and supporting their use in clinical practice for better disease management and patient outcomes.

## 1. Introduction

Ovarian tumors are rarely diagnosed in the pediatric age group, with an estimated incidence starting from 0.4 per 100.000 girls per year during infancy to 25–30 per 100.000 girls per year at the age of 18. Around 10–30% of all pediatric ovarian masses in the USA are malignant [1]. The rate of malignancy increases from birth (18% malignant) until the age of 6–7 years (30% malignant), and then decreases significantly afterwards (less than 10% at the age of 14) [2]. According to the current World Health Organization (WHO) classification, children’s ovarian tumors are categorized into four main histopathological groups. Germ cell tumors (GCTs) represent the most common group, accounting for 60–75% of cases [3,4]. Epithelial cell tumors (ETs) represent the second group (10–20%), while sex cord stromal tumors (SCSTs) account for up to 10% of cases [3]. Finally, a fourth, less common group includes solid or hemolymphoid cancers and rare tumors that start or spread in the ovary [5,6]. 

Specimens obtained from biopsy and surgical procedures should be examined by a qualified pediatric specialist pathologist because there is a substantial risk of misdiagnosis due to the rarity of ovarian malignant tumors in non-adult patients. Only several clinical practice guidelines recommend, with high priority, the use of immunohistochemistry and molecular tests in order to clarify potential diagnostic challenges and validate the diagnosis. Diagnoses of difficult cases of GCTs may be guided by a panel of immunohistochemical (IHC) markers (SALL4, OCT3/4, PLAP, D2-40, NANOG, SCFR, AFP, and Glypican-3), in addition to isochromosome 12 (12 p-fluorescent in situ hybridization) [7,8,9,10,11,12]. 

The main objectives of this study were to examine the clinical and pathological characteristics of ovarian tumors in children, to analyze the expression patterns of specific IHC markers (SALL4 and OCT3/4) in different types of ovarian tumors, and to analyze the association between the expression of these markers with classical clinicopathological features and survival outcomes. 

## 2. Results

### 2.1. Patients’ Clinical and Pathological Characteristics

**Figure 1:**
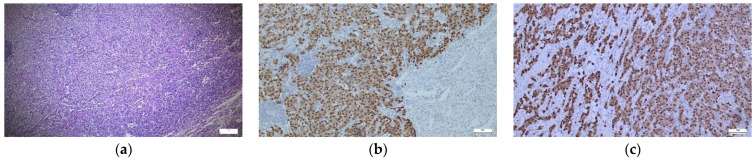
(**a**–**c**) Germinal tumor—Hematoxylin–Eosin and SALL4/OCT3/4 immunostaining expression in tumor cells. (**a**) Small hyperchromic tumor cells mixed with large cells with obvious nucleus and nucleoli, some binucleate (×5). (**b**) OCT3/4—strong positive nuclear expression in tumor cells (×10). (**c**) SALL4—uniform positive nuclear expression in tumor cells (×10).

**Figure 2:**
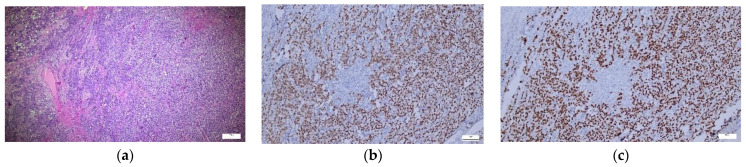
Dysgerminoma—Hematoxylin–Eosin and SALL 4/OCT3/4 immunostaining expression in tumor cells. (**a**) Tumor cells, of medium and large size, polygonal appearance with large nuclei, prominent nucleoli, atypical mitoses, tumor stoma with lymphoid infiltrates, areas of necrosis, and hemorrhage (×5). (**b**) OCT3/4—diffuse, positive nuclear expression in tumor cells with strong intensity (×10). (**c**) SALL4—positive, homogeneous staining nuclear expression in tumor cells (×10).

The study cohort included a total number of 64 patients, diagnosed with ovarian tumors, from January 2007 to January 2023. A total of 43.8% of the patients were under 12 years old, and 56.3% were between 12 and 18 years old (Table 1). The majority of patients came from rural areas (62.5%). In terms of the year of diagnosis, most patients were diagnosed in 2019 (14.1%), 2020 (14.1%), or 2011 (10.9%). The most common symptoms were abdominal pain (89.1%) and the presence of palpable masses (32.8%). Vomiting (14.1%) was more frequent in patients aged 0–11 years (25%) than in patients aged 12–18 years (5.6%) (*p* = 0.035). An amount of 28.1% of the patients underwent chemotherapy (CHT) (82.4% with a single protocol, 17.6% with two protocols, with a mean number of sessions of 6.82 ± 2.72, median = 8). The majority of tumors were GCTs (84.4%) (Figure 1a–c), with predominantly mature teratomas (51.6%—33 cases) or immature teratomas (20.3%—13 cases). A smaller percentage of GCTs were DSG (6.3%—four cases) (Figure 2a–c), mixed tumors (3.1%—two cases), and only one yolk sac tumor (YST) and embryonal carcinoma (EC) (1.6% each). ETs were found in 10.9% of cases and had a relatively uniform distribution, with two cases of adenocarcinoma (3.1%), two borderline tumors with serous epithelium (3.1%), mucinous borderline tumors (EMBTs) (3.1%), and one case of a serous tumor (1.6%). Among SCSTs (4.7%), there were two cases of adult-type granulosa cell tumors (1.6%), juvenile-type granulosa cell tumors (1.6%), and one case of mixed sex cord stromal tumors (1.6%). In terms of staging, the majority were classified as pT1a (46.9%), pT2a (32.8%), pT1a (46.9%), pT3a (4.7%), and pT1c3, pT2b, pT3b, and pT3c (1.6% each). Most patients had a favorable outcome (96.9%), 3.1% had metastases at admission, 6.3% had subsequent metastases, one patient had a recurrence, and two patients (3.1%) died. 

### 2.2. Patients’ Distribution Based on SALL4/OCT3/4 Immunostaining and Tumor Type and Subtypes

The distribution of patients based on IHC positive staining and age show that 29.7% of tumors were positive for SALL4 (6 patients in the 0–11 age group and 13 patients in the 12–18 age group), and 6.3% were positive for OCT3/4 (with an equal distribution across age groups). There were no statistically significant differences observed between histological type groups in marker values according to Fisher’s exact test (Table 2). In terms of patient distribution based on the value of the investigated markers and sub-type tumor classification, the results showed that the frequency of SALL positivity was significantly different (***p* < 0.001**). Bonferroni-corrected Z tests indicated that patients with adenocarcinoma (10.5% vs. 0%), DSG (21.1% vs. 0%), mixed GCTs (10.5% vs. 0%), and immature teratoma (36.8% vs. 13.3%) were significantly more frequently associated with SALL4 positivity, whereas patients with mature teratoma (73.3% vs. 0%) were significantly more frequently associated with SALL4 negativity. In relation to tumor sub-type classification, the frequency of OCT3/4 immunostaining was significantly different (*p* = 0.002), and Bonferroni-corrected Z tests indicated that patients with DSG (75% vs. 1.7%) and mixed GCTs (25% vs. 1.7%) were significantly more frequently associated with OCT3/4 positivity, while patients with mature teratoma (55% vs. 0%) were significantly more frequently associated with OCT3/4 negativity.

After analyzing the final IHC scores of the markers that were used to classify the tumors, the SALL4 final score was 4.63 ± 0.95 (mean ± SD), with a median (IQR) of 5 (4–5), and the OCT3/4 final score was 5.25 ± 0.5 (mean ± SD), with a median (IQR) of 5.25 (5–5.75). High immunostaining expression for SALL4 was observed primarily in YST (score 6), followed by mixed GCTs with components of YST (mean ± SD = 5.5 ± 0.7), DSG (mean ± SD = 5.25 ± 0.25), and adenocarcinoma (score 5), with lower expressions seen in EC (score 3), EMBTs (score 3), and serous borderline tumors (SBTs, score 4). OCT3/4 immunostaining was positive exclusively in GCTs, with high expressions in mixed GCTs (score 6) and DSG (score 5).

The results regarding the distribution of patients based on the IHC and tumor staging showed that the frequency of SALL4 positivity was significantly different in relation to tumor subtypes (***p* = 0.002**). According to Bonferroni-corrected Z tests, patients with tumors at stage pT3a (15.8% vs. 0%) were significantly more frequently associated with SALL4 positivity, whereas patients with tumors at stage pT1a (57.8% vs. 21.1%) were significantly more frequently associated with SALL4 negativity. Additionally, the frequency of OCT3/4 positivity was significantly different in relation to tumor subtypes (***p* = 0.037**). Bonferroni-corrected Z tests showed that patients with tumors at stage pT3a (25% vs. 3.3%) or pT3b (25% vs. 0%) were significantly more frequently associated with OCT3/4 positivity.

### 2.3. Patients’ Distributions Based on IHC Staining and the Level of STM

We observed that patients with elevated AFP values were significantly more frequently associated with positive SALL4 values (41.2% vs. 9.7%), while patients with normal AFP values were significantly more frequently associated with negative SALL4 values (90.3% vs. 58.8%). Fisher’s exact test showed that there were statistically significant differences between AFP values and SALL4 (***p* = 0.022**), and LDH values and SALL4 (***p* = 0.003**), respectively. We noticed that patients with elevated LDH values significantly more frequently had positive SALL4 staining (62.5% vs. 5%).

### 2.4. Patients’ Distributions Based on IHC Markers and CHT

Fisher’s exact test revealed no statistically significant differences between groups in the distribution of patients based on IHC markers and the number of CHT protocols required (Table 3). However, Student’s *t*-test (***p* = 0.001**) revealed significant statistical differences in the number of CHT sessions required and the positivity of the investigated markers, only in the SALL4 group. Patients with positive SALL4 had a significantly higher number of sessions (8.08 ± 1.93) compared to patients with negative SALL4 (3.8 ± 1.79). 

### 2.5. Patient’s Distributions Based on IHC Markers and Overall Outcomes 

The differences between groups of patients based on the immunostaining markers and their outcome, recurrence, and death were not statistically significant according to Fisher’s exact tests for any of the markers (*p* > 0.05). As a result, there were no significant associations between outcome, recurrence, mortality, or the markers analyzed in the study. The data in Table 4 represent the distribution of patients based on IHC markers and the presence of subsequent metastases. The results showed that the markers were significantly associated with an increased frequency in subsequent metastases: SALL4—21.1% vs. 0% (***p* = 0.006**) and OCT3/4—50% vs. 3.3% (***p* = 0.017**), indicating that patients who had subsequent metastases were significantly more frequently associated with the presence of at least one of the markers analyzed in the study.

We calculated univariate Cox hazard models (HR 95% C.I.) to predict the values of biomarkers on the risk of subsequent metastasis. The results indicated that OCT3/4 positivity (***p* = 0.017**) significantly influenced the risk of subsequent metastasis, while SALL4 did not significantly influence the prediction (*p* = 0.275). Patients with positive OCT3/4 had a 10.912 times higher risk of subsequent metastasis (95% C.I.: 1.528–77.9). A multivariable model could not be performed due to the small number of cases with subsequent metastases.

**Figure 3:**
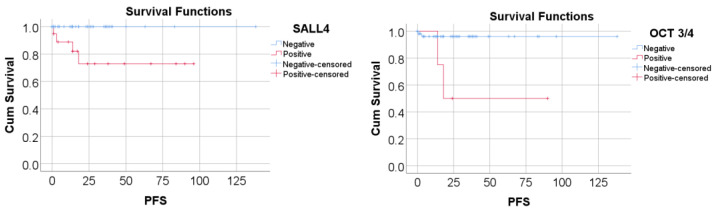
Kaplan–Meier curve: comparison of PFS based on SALL4/OCT3/4 immunostaining.

The mean overall survival (OS) period was 132.3 months (95% C.I.: 124.7–140). The mean progression-free survival (PFS) period was 126.6 months (95% C.I.: 115.7–137.4). The overall survival rate was 96.87%. Figure 3 shows a comparison of PFS based on the analyzed markers’ immunostaining. According to Tarone–Ware tests, patients with positive IHC markers had a significantly shorter PFS compared to patients with negative markers: positive SALL4: mean PFS = 72.78 (95% C.I.: 52.84–92.72) vs. negative SALL4: mean PFS = 138 (***p* = 0.003**); positive OCT 3/4: mean PFS = 53 (95% C.I.: 16.71–89.28) vs. negative OCT 3/4: mean PFS = 132.64 (95% C.I.: 125.35–139.93) (***p* = 0.008**). 

## 3. Discussion

There are only a few limited worldwide reports regarding molecular markers in pediatric ovarian tumors, and it is important to identify their potential in this rare but clinically important pediatric cancer. Our study provides novel insights into the roles of SALL4 and OCT3/4 in pediatric ovarian tumors, an area that has not been extensively studied. These markers have been previously linked to ovarian tumors, but our research focused on their implications in a pediatric context. We conducted a search through PubMed for published data using a similar IHC panel for pediatric and adult ovarian tumors, covering the period from 2000 to 2023 (Table A1). This systematic research revealed 17 original studies eligible for assessing the use of one or more IHC markers for ovarian tumors [13,14,15,16,17,18,19,20,21,22,23,24,25,26,27,28,29,30]. Specifically, we identified only six reports with pediatric/adolescent populations [16,17,24,25,27,29].

The inclusion of molecular markers in the management of ovarian tumors, as predictive factors, is crucial and necessary for an accelerated risk stratification and personalized treatment approach [31]. Cyclin D1 and Ki-67 levels have been shown to be sensitive and specific in predicting patient outcomes in ovarian tumors. They have also been useful in the histological classification of teratomas, and higher p27 levels are linked to earlier stages of the tumor [32]. An increased expression in matrix metalloproteinases (MMPs) has been correlated with cancer invasion, metastatic potential, and a negative prognosis. High MMP3 expression has been noted in advanced and aggressive tumors compared to that in early-stage tumors with lower malignant potential [33]. In ovarian tumors, MMP2 and MMP13 expression have been associated with lower overall survival, while MMP9, MMP10, MMP12, and MMP25 positivity have been correlated with a positive prognosis [34]. MMPs are essential in the metastatic process by increasing cellular motility and regulating the epithelial–mesenchymal transition. The activation of STAT3 stimulates the expression of MMP1, MMP2, and MMP9 [35]. Ezrin and p130Cas are structural proteins with an important role in signaling pathways, promoting tumor dissemination. In vitro results coincide with clinical specimen analysis, suggesting that in ovarian cancer, the role of ezrin in disease progression is more pronounced than that of p130Cas [36]. Many pediatric germ cell tumors express P-glycoprotein, a membrane-bound protein that can decrease the response to adjuvant chemotherapy, and its expression is associated with treatment-refractory tumors [37]. 

SALL4 is one of the four members of the SALL family of stem cell transcription factors and is a zinc protein essential for the development of embryonic stem cells and the self-renewal of embryonic stem cells [38]. SALL4 has different mechanisms and regulatory functions in tumors and human hematopoiesis. SALL4 overexpression promotes proliferation, development, invasion, and migration in cancers through activation of the Wnt/β-catenin, PI3K/AKT, and Notch signaling pathways [39]. The activation of the STAT3 pathway is well known to be associated with tumor progression and metastasis in a number of cancers, including ovarian cancer [40].

OCT3/4 is a member of the POU domain family of transcription factors and has been shown to play an important role in maintaining self-renewal and pluripotency in embryonic stem cells (ESCs) [41]. The “Yamanaka” transcriptional factors”—OCT3/4, SOX-2, c-Myc, and KLF4—have the capacity to propagate indefinitely in vitro and differentiate into all somatic cell types when they receive signals from the environment, giving them significant potential for regenerative therapies [42]. OCT3/4 and its activation targets have been overexpressed in the stem cells of various tumors and have been associated with tumor pathogenesis, development, and poor prognosis. The co-expression of OCT3/4 and CD133 has been used in clinical staging and thr risk stratification of tumors and can provide essential principles for treatment planning and patient monitoring [43].

In a recent study, Atilgan et al. highlighted ARID1A and SALL4 expression in ovarian seromucinous tumors (SMTs), including borderline tumors (SMBTs) and endometrioid carcinoma with mucinous differentiation (SMC). Of 26 SMC cases, only one showed focal SALL4 positivity. All 12 SMTs were positive for ER, PR, PAX8, and CK7 and negative for WT1, CK20, CDX2, and CEA [13]. In our research, we observed that SALL4 varied significantly by tumor type, with higher expression in adenocarcinoma, DSG, mixed GCTs, and immature teratoma. The frequency of SALL4 positivity was significantly different across tumor subtypes. 

Yang et al., investigated 91 cases of serous ovarian carcinoma (SOC) and observed that the SALL4 protein was highly expressed in SOC tissues and positively associated with an advanced FIGO stage, high histological grade, lymph node involvement, distant metastasis, recurrence, and the death of SOC [14]. Our research demonstrated that SALL4 immunostaining was associated with advanced tumor stages and a subsequent metastasis. However, we did not observe significant associations between SALL4 expression and recurrence or mortality in the pediatric population. Patients with positive SALL4 immunostaining had a significantly higher number of CHT sessions compared to patients with negative SALL4. We discovered that SALL4 is a highly sensitive and specific marker for primitive ovarian GCTs, corroborating the results of Cao et al., who found SALL4 to be more reliable than AFP, glypican-3, CK7, and EMA in distinguishing YSTs from ovarian clear cell carcinoma [15]. Similar findings were reported in 15 pediatric cases, where SALL4 expression was seen in all YSTs, with a better advantage over glypican-3 and AFP in diagnosing pediatric YSTs [16].

SCSTs may be misinterpreted as YSTs when they exhibit a “reticular” growth pattern and contain high mitotic activity. To distinguish these two tumors, some authors have performed IHC stains for SALL4 and steroidogenic factor-1 (SF-1). All YSTs were positive for SALL4 (100%) and negative for SF-1 (100%). In contrast, all the SCSTs were positive for SF-1 and negative for SALL4 (100%). The difference was significant, and their result indicated that these two markers could be used as a pair of markers to differentiate these two tumors in a challenging situation [17]. Our results support the use of SALL4 as a diagnostic tool in pediatric ovarian tumors and as a reliable marker for identifying YSTs. 

To investigate the correlations among OCT4, Notch1, and DLL4 between patients with epithelial ovarian cancer (EOC) and benign ETs, a study was conducted by Lan et al. [18]. Their finding suggested that the expressions of OCT4, Notch1, and DLL4 in EOC were significantly correlated with tumor differentiation, stage, and lymph node metastasis. The over-expressions of OCT4, Notch1, and DLL4 were associated with a poor prognosis, and the survival rate was significantly lower in positive IHC cases than in the negative ones [18]. Our study found significant expressions of OCT3/4, especially in cases of DSG and mixed GCTs. Similar to Lan et al.’s [18] results, OCT3/4 expression was correlated with advanced tumor stages and had a higher risk of subsequent metastasis, highlighting the potential role of OCT3/4 as a prognostic marker in both pediatric and adult ovarian tumors. Similar findings were reported by Zhang et al., suggesting that OCT4 overexpression was associated with a higher FIGO stage and histological grade in serous adenocarcinoma [19]. Consistent with their results, this similarity with our findings reinforces the importance of OCT3/4 as a marker for tumor aggressiveness across different age groups and tumor types. 

Cheng et al., analyzed the expression of OCT4 in both DSG and non-DSG ovarian cancers. All cases of DSG and gonadoblastoma were positive for OCT4 with strong nuclear staining. All non-DSG tumors were negative for OCT4, except for four cases of clear cell adenocarcinoma of the ovary. Results from this study suggested that OCT4 may aid in the detection of metastatic DSG in extraovarian sites and in distinguishing DSG from other primary and metastatic tumors in the ovary [20]. This finding was consistent with our results that OCT3/4 was expressed in GCTs, especially in DSG, while it was not staining in other types of tumors. 

Expressions of the chromosomal 12 p anomalies OCT4, CD30, SOX2, and glypican-3 were analyzed in a series of six mixed ovarian GCTs with a component of EC and compared to four cases of mixed GCTs that were originally mistaken for EC [21]. Two cases initially diagnosed as EC were negative for OCT3/4 and CD30 and positive for glypican-3, leading to their reclassification as YSTs. In contrast, two other cases initially diagnosed as EC showed positive OCT3/4 staining and negative CD30 staining and were reclassified as immature teratoma with neuroectodermal differentiation. It was found that chromosome 12p fluorescence in situ hybridization combined with IHC staining of OCT4, CD30, and glypican-3 was valuable for confirming the diagnosis of ovarian EC [21]. Our findings on OCT3/4 immunoexpression aligned with the reclassification insights provided by the literature study, emphasizing the diagnostic value of OCT3/4 in identifying and distinguishing specific ovarian tumor subtypes. 

In another investigation, Chang et al., observed OCT3/4 positive staining in four of nine adult granulosa cell tumors, and in a small number of surface-epithelial stromal tumors, SOX2 and/or OCT3/4 were variably positive. SOX2 and D2-40 differentiated between DSG and EC. NANOG was able to distinguish between either of these two tumors and non-GCTs [22]. Both studies emphasized the role of OCT3/4 in tumor classification and prognosis, underscoring its utility in clinical management. Salonen et al. evaluated the prognostic value of STM, AP-2, and OCT3/4 expression in malignant GCT. Their results highlighted that elevated preoperative levels of serum CA-125 in positive immunostaining tissues were prognostic of progressive disease [23]. In our research, we observed that patients with elevated AFP and LDH values were significantly more frequently associated with positive SALL4 values. 

In another study, expressions of OCT3/4, PAX6, and CD56 were evaluated in immature neuroepithelium in child and adult ovarian teratoma. OCT3/4 was expressed in seven cases with grade 3 immature teratoma and only in two cases with grade 2 immature neuroepithelium. OCT3/4 was negative in grades 1 or 2 in the cases. The results from this study suggested that OCT3/4 was detected only in high-grade immature teratomas and that might serve as a promising tool for the clinical management of this disease [24]. In our study, OCT3/4 was negative for all cases of immature teratoma, showing that OCT3/4 positivity was a strong indicator of more aggressive and advanced diseases, particularly in GCTs. 

In an analysis with pediatric GCTs, all YSTs expressed AFP and SALL4 staining, with GATA4-positive immunostaining in ~90%. SALL4 was found in immature teratoma, whereas the majority of this type of tumor was positive for SOX2 and PDPN expression. OCT4, SALL4, and PDPN were all expressed in DSG. They revealed an unrecognized pathogenic link between AFP and SALL4 in YSTs, indicating varying differentiation statuses. Their findings suggested a similar antigen expression pattern between pediatric and adult GCTs, despite their development along distinct development pathways. OCT3/4 and AP-2γ were negative in YSTs, MTs, and SCSTs, but had high expression in DSG and a few immature teratomas [25]. The results underlined our reports and highlighted the importance of SALL4 and OCT3/4 as biomarkers in pediatric GCTs. The consistent findings across studies validate SALL4 as a marker for YSTs and immature teratomas and OCT3/4 for DSG. Additionally, our study enhances the understanding of these biomarkers by highlighting their prognostic value, thereby contributing to more effective clinical management of pediatric ovarian tumors. 

According to a large series of 3215 tumors investigated by Miettinen et al. for SALL4 expression, SALL4 was consistently expressed in all GCTs, except some components of mature teratomas, and were detected in almost 20% of cases in non-GC (ovarian serous carcinoma). They supported the hypothesis that absence of additional pluripotency factors, OCT4 and NANOG, in SALL4-positive non-GCT may also provide diagnostic support [26]. Another study investigated the frequency of CD117, CD133, SALL4, OCT4, TCL1, and glypican-3 marker expressions in ovarian malignant GCTs and reported that CD117 is a useful tool in diagnostics for DSG and YSTs. SALL4 has a greater sensitivity and specificity for YSTs compared to glypican-3. Additionally, SALL4 and OCT3/4 are valuable in distinguishing YSTs from DSG [27]. Both studies highlighted SALL4 as a consistent marker for GCTs, corroborating our findings of strong SALL4 expression in YSTs and immature teratomas. 

Some authors have investigated the utility of SALL4 as a potential diagnostic marker for metastatic GCTs. SALL4 was found to be strongly positive in all metastatic DSG, EC, and YSTs. These results identified SALL4 as a sensitive and highly specific marker for metastatic GCTs, particularly for detecting metastatic YSTs [28]. 

There were no significant associations between the outcome, recurrence, mortality, or the markers analyzed in the study. OCT3/4- and SALL4-positive staining were significantly associated with an increased frequency in subsequent metastases. OCT3/4 positivity significantly affected the risk of subsequent metastasis, whereas SALL4 did not significantly influence the prediction. Patients with positive markers had a significantly shorter PFS compared to patients with negative markers.

Target therapy: Due to its high expression in tumor cells, SALL4 has represented an ideal target for cancer treatment [38], and multiple clinical trials and treatment studies have been undertaken to inhibit its expression [44]. The activation of the STAT3 signaling pathway has been associated with tumor progression and metastatic potential in numerous types of cancer, including ovarian cancer. Therefore, STAT3 has been considered a potential ideal target for ovarian cancer treatment. 

Napabucasin has been used in monotherapy or in combination with MG-132 to highlight its antitumor activity by inhibiting the STAT3 signaling pathway. Napabucasin has demonstrated significant tumor inhibitory effects against ovarian cancer cells (SKOV-3) and induced autophagy in these cells, contributing to the inhibition of cell proliferation and providing a potential treatment strategy. The combination of napabucasin with MG132 has shown significant synergistic effects in inhibiting tumor cell proliferation, possibly by inducing apoptosis through mitochondrial-dependent mechanisms [40]. The potential association between refractory or cisplatin-resistant tumors and cancer stem cell markers has been investigated in cisplatin-resistant yolk sac ovarian tumors to determine targeted therapy and overcome resistance. In NOY-1 CisR ovarian cells, treatment with salinomycin or tunicamycin has been shown to reduce cancer stem cell markers and provide therapeutic benefits. NOY-1 CisR cells demonstrated higher sensitivity to treatment with salinomycin and tunicamycin compared to control ovarian cells. Combined therapy with napabucasin amplified cisplatin toxicity [45]. 

iPSC-derived NK cell therapy has been successful in treating solid tumors, including ovarian cancer. iPSC-NK cells were used to treat mice inoculated with ovarian cancer cells (MA148), and the median survival improved from 73 to 98 days, suggesting the significant efficacy of the therapy in extending survival in the presence of ovarian cancer. Moreover, these iPSC-NK cells were found in the peritoneal cavity of mice and markedly inhibited tumor growth, indicating their potential for efficiently controlling the progression of solid tumors [42]. Non-coding nucleic acids are DNA or RNA molecules that do not encode proteins but play a crucial role in regulating gene expression and other cellular processes. Types of oligonucleotide-based therapies include siRNA, shRNA, ODN-decoy, and antisense oligonucleotide (ASO), which have demonstrated significant therapeutic potential [35]. AZD9150 (danvatirsen) is an antisense oligonucleotide (ASO) inhibitor of STAT3 that has shown clinical activity in several phase I/II clinical studies. Combined therapies involve the use of STAT3-specific ASO simultaneously with radiotherapy, chemotherapy, or immunotherapy. In ovarian cancer, clinical trials with AZD9150 were stopped due to the inability to find eligible patients to participate in the study [42].

Clinical correlations with marker expression require multiple future studies to highlight the possibility of developing new treatment strategies focused on the expression of SALL4, OCT3/4, and their signaling pathway inhibition methods.

## 4. Materials and Methods

### 4.1. Patients

We conducted a retrospective study and identified patients with ovarian tumors who were admitted to the “Saint Mary” Emergency Hospital in Iasi, Romania, from January 2007 to January 2023, utilizing the ICD-10 coding system. We examined their medical records in order to obtain epidemiological and clinical data, information on staging imaging, STM levels, details of surgical procedures, CHT, clinical outcomes, and the pathology’s report. We classified the patients into two age cohorts: 0–11 years and 12–18 years. The tumors had been assessed based on the criteria established by the WHO in 2020. The clinical stage was established according to the macroscopic aspect of primary tumors based on the guidelines of the International Federation of Gynecology and Obstetrics (FIGO).

### 4.2. Immunohistochemical Analysis and Assessment Protocol

The immunohistochemical exam was performed using two monoclonal nuclear antibodies: anti-SALL4 and anti-OCT3/4 (SALL4 antibody, isotype IgG1/K, clone 6E3, source mouse, dilution 1:100, antigen retrieval—pH 9, BioSB; OCT3/4, clone N1NK, source mouse, dilution 1:100, antigen retrieval—pH 6, Novocastra) after pretreatment with enzyme proteinase K for 5 min. For immunoreaction detection, we used an UltraVision Quanto Detection System HRP DAB (ThermoFisher Scientific, Fremont, CA, USA). Controls were included in each run. Two independent pathologists performed the evaluation of the markers. The immunostaining patterns of our antibodies were limited only to nuclear expression. Membranous or cytoplasmic staining was not considered positive. A semi-quantitative four-tiered scoring system was used to evaluate the staining intensity as: 0 (no staining), 1 (faint yellow), 2 (brown–yellow), and 3 (dark yellow). The extent of staining used to evaluate the percentage of positive tumor cells was scored as 0 (less than 5%), 1 (5–25% positive cells), 2 (26–75% positive cells), and 3 (more than 76% positive cells) [19]. The final immunoreactivity score was computed by summing the intensity and the extent of staining scores, respectively. As a result, the expression categories were finally defined as: negative (0), + (1–2), ++ (3–4), and +++ (5–6) [19].

### 4.3. Statistical Analysis

All the data from the study were analyzed using IBM SPSS Statistics 25 and illustrated using Microsoft Office Excel/Word 2013. Independent quantitative variables with normal distribution were tested between two independent groups using Student’s *t*-test, while independent quantitative variables with non-parametric distribution were tested between groups using the Mann–Whitney U test. Qualitative variables were expressed as absolute frequencies and percentages, and differences between groups were tested using Fisher’s exact test. Z tests with Bonferroni correction were used to analyze the results obtained in the contingency tables. Kaplan–Meier curves were used to illustrate and calculate the OS and PFS periods in the analyzed groups. Tarone-Ware tests were used for the comparison of OS and PFS periods between age groups. Univariable Cox regression models for hazard estimation were used to calculate the risk of subsequent metastasis occurrence determined by the presence of the analyzed markers in the study. 

Our study had several limitations. The sample size was relatively small, which may have affected the generalizability of the findings. The study was retrospective in nature, which introduces inherent biases. Additionally, there may have been a selective bias because our institution is not a national referral medical center for ovarian tumors. It is important to note that we do not discuss extensive long-term follow-up due to the fact that patients are treated in our hospital only until 18 years of age; after this age, they are referred to an adult oncological center. Furthermore, we lack access to a national cancer registry, which is critical for continued monitoring in adult care settings and extensive long-term follow-up.

## 5. Conclusions

This study highlighted the importance of immunostaining for valuable biomarkers, SALL4 and OCT3/4, in pediatric ovarian tumors. It demonstrated significant correlations between these biomarkers and tumor type, as well as their ability to predict treatment response, metastasis, and prognosis. The results underscored the need for further research and clinical trials to validate these findings and explore the potential of targeted therapies based on SALL4 and OCT3/4 expression in improving outcomes for pediatric ovarian tumor patients. This study fills in an important gap in the current literature by adding new information about the molecular features and clinical effects of SALL4 and OCT3/4 in pediatric ovarian tumors.

## Figures and Tables

**Table 1:** Characteristics of the patients analyzed in the study, reported by age.

Parameter/Age	Total	0–11 Years	12–18 Years	*p*
***Number of patients***	64 (100%)	28 (43.8%)	36 (56.3%)	-
***Origin (No., %)***				
Rural	40 (62.5%)	23 (82.1%)	17 (47.2%)	0.005 *
Urban	24 (37.5%)	5 (17.9%)	19 (52.8%)	
***WHO Classification (No., %)***				
Epithelial tumors	7 (10.9%)	2 (7.1%)	5 (13.9%)	0.665 *
Germinal cell tumors	54 (84.4%)	25 (89.3%)	29 (80.6%)	
Sex cord stromal tumors	3 (4.7%)	1 (3.6%)	2 (5.6%)	
***Serum tumor markers (STM) (No., %)***				
Elevated AFP	10 (20.8%)	5 (26.3%)	5 (17.2%)	0.487 *
Elevated hCG	15 (31.9%)	8 (44.4%)	7 (24.1%)	0.202 *
Elevated LDH	6 (21.4%)	1 (12.5%)	5 (25%)	0.640 *
***Location (No., %)***				
Left	29 (45.3%)	12 (42.9%)	17 (47.2%)	0.914 *
Right	32 (50%)	15 (53.6%)	17 (47.2%)	
Bilateral	3 (4.7%)	1 (3.6%)	2 (5.6%)	
***Chemotherapy (No., %)***	17 (26.6%)	7 (25%)	10 (27.8%)	1.000 *
***Outcome (No., %)***				
Unfavorable	2 (3.1%)	0 (0%)	2 (5.6%)	0.500 *
Favorable	62 (96.9%)	28 (100%)	34 (94.4%)	
***Metastasis at admission (No., %)***	2 (3.1%)	1 (3.6%)	1 (2.8%)	1.000 *
***Subsequent metastasis (No., %)***	4 (6.3%)	1 (3.6%)	3 (8.3%)	0.625 *
***Recurrence (No., %)***	1 (1.6%)	0 (0%)	1 (2.8%)	1.000 *
***Death (No., %)***	2 (3.1%)	0 (0%)	2 (5.6%)	0.500 *
***Follow-up period***				
Mean ± SD	27.42 ± 28.14	35.04 ± 35.23	21.5 ± 19.64	0.326 **
Median (IQR)	18 (5.25–38)	27 (2.5–59.75)	15 (7.25–34)	
OS (months) (Mean (95% C.I.))	132.3 (124.7–140)	138	89.4 (80.8–98.1)	0.227 ***
PFS (months) (Mean (95% C.I.))	126.6 (115.7–137.4)	131.1 (117.9–144.2)	85.7 (74.3–97)	0.435 ***

* Fisher’s exact test, ** Mann–Whitney U test, *** Tarone–Ware test.

**Table 2:** Distribution of patients based on SALL4/OCT3/4 immunostaining and tumor types and subtypes.

*Tumor Type* *SALL4*	*SALL4 Negative*		*SALL4 Positive*		p *
	Nr.	%	Nr.	%	
ETs	3	6.7%	4	21.1%	0.162
GCTs	39	86.7%	15	78.9%	
SCSTs	3	6.7%	0	0%	
***Tumor Type*** ***OCT 3/4***	***OCT 3/4 Negative***		***OCT 3/4 Positive***		p *
	**Nr.**	**%**	**Nr.**	**%**	
ET	7	11.7%	0	0%	1.000
GCTs	50	83.3%	4	100%	
SCSTs	3	50%	0	0%	
***Tumor Subtype*** ***SALL4***	***SALL4 Negative***		***SALL4 Positive***		p *
	**Nr.**	**%**	**Nr.**	**%**	
Adenocarcinoma	**0**	**0%**	**2**	**10.5%**	**<0.001**
Dysgerminoma	**0**	**0%**	**4**	**21.1%**	
EC	0	0%	1	5.3%	
EMBTs	1	2.2%	1	5.3%	
Mixed GCTs	**0**	**0%**	**2**	**10.5%**	
Mixed SCST	1	2.2%	0	0%	
Pure SCST	2	4.4%	0	0%	
Serous Cyst	1	2.2%	0	0%	
SBT	1	2.2%	1	5.3%	
Immature Teratoma	**6**	**13.3%**	**7**	**36.8%**	
Mature Teratoma	**33**	**73.3%**	**0**	**0%**	
YST	0	0%	1	5.3%	
***Tumor Subtype*** ***OCT ¾***	***OCT 3/4 Negative***		***OCT 3/4 Positive***		p *
	**Nr.**	**%**	**Nr.**	**%**	
Adenocarcinoma	2	3.3%	0	0%	**0.002**
Dysgerminoma	**1**	**1.7%**	**3**	**75%**	
EC	1	1.7%	0	0%	
EMBT	2	3.3%	0	0%	
Mixed GCT	**1**	**1.7%**	**1**	**25%**	
Mixed SCST	1	1.7%	0	0%	
Pure SCST	2	3.3%	0	0%	
Serous Cyst	1	1.7%	0	0%	
SBT	2	3.3%	0	0%	
Immature Teratoma	13	21.7%	0	0%	
Mature Teratoma	**33**	**55%**	**0**	**0%**	
YST	1	1.7%	0	0%	

* Fisher’s exact test.

**Table 3:** Distribution of patients based on SALL4/OCT3/4 and CHT.

*Protocol No./SALL4*		*SALL4 Negative*		*SALL4 Positive*		p *
		Nr.	%	Nr.	%	
One protocol		5	100%	9	75%	0.515
Two protocols		0	0%	3	25%	
***Protocol No./OCT 3/4***		***OCT 3/4 Negative***		***OCT 3/4 Positive***		p *
		**Nr.**	**%**	**Nr.**	**%**	
One protocol		12	92.3%	2	50%	0.121
Two protocols		1	7.7%	2	50%	
* Fisher’s Exact Test						
Comparison of the CHT Sessions Based on the Marker Immunostaining						
***Sessions No.* /SALL4**	***Mean ± SD***			***Median (IQR)***	***Mean Rank***	p *
Negative (*p* = 0.238 **)	3.8 ± 1.79			4 (2.5–5)	-	**0.001**
Positive (*p* = 0.064 **)	8.08 ± 1.93			8 (6.5–10)	-	
***OCT 3/4***	***Mean ± SD***			***Median (IQR)***	***Mean Rank***	p ***
Negative (*p* = 0.370 **)	6.15 ± 2.73			6 (4–8.5)	7.77	0.079
Positive (*p* = 0.024 **)	9 ± 1.15			9 (8–10)	13.00	

* Student’s *t*-test, ** Shapiro–Wilk Test, *** Mann–Whitney U test.

**Table 4:** Distribution of patients based on SALL4/OCT3/4 and subsequent metastasis.

*Metastasis/SALL4*	*SALL4 Negative*		*SALL Positive*		p *
	Nr.	%	Nr.	%	
Absent	45	100%	15	78.9%	**0.006**
Present	0	0%	4	21.1%	
***Metastasis/OCT 3/4***	***OCT 3/4 Negative***		***OCT 3/4 Positive***		p *
	**Nr.**	**%**	**Nr.**	**%**	
Absent	58	96.7%	2	50%	**0.017**
Present	2	3.3%	2	50%	

* Fisher’s exact test.

## Data Availability

Data are contained within the article.

## Appendix A

**Table A1:** Chronological summary of published data using a similar IHC panel for ovarian tumors.

Studies	Cases N.	C/A u	Year	IHC	GCT							ET	SCST
					MT	IT	YST	DG	EC	MGC	Cc		
Atilgan et al. [13]	38	A	2023	+ SALL 4									1/ 38
**Wu et al.** **[16]**	**15**	**C**	**2020**		**1/** **10**		**5/** **5**						
Yang et al. [14]	91	A	2016									53	
**Bai et al.** **[17]**	**25**	**A, C**	**2013**			**1/** **1**							**0/** **24**
Cao et al. [15]	251	A	2009		0/ 12	11/ 15	16/ 16	18/ 18	6/ 6	13/ 13		3/ 111	0/ 42
Yu et al. [18]	207	A	2016	+ OCT 3/4								124	
**Talebagha et al.** **[29]**	**5**	**C**	**2013**					**3/** **3**	**1/** **1**	**2/** **2**			
Cheng et al. [21]	10	A	2010							6/6 2/4			
**Abiko et al.** **[24]**	**18**	**A, C**	**2010**			**9/** **18**							
Zhang et al. [19]	420	A	2010									333	
Chang et al. [22]	88	u	2009		0/ 5	0/ 9	0/ 4	7/ 9	1/ 1	3/ 3		1/ 32	4/ 25
Salonen et al. [23]	30	A	2008			0/ 5	1/ 4	3/ 5		1/ 1			
Cheng et al. [20]	144	A	2004		0/ 14		0/ 4	35/ 35					
**Mosbech et al. [25]**	**9**	**C**	**2014**	**SALL4** **OCT3/4** **PLAP**	**0/1** **0/1** **0/1**	**1/1** **1/1** **0/1**	**3/3** **0/3** **1/3**						**0/4**
Miettinen et al. [26]	95	u	2014	SALL4 OCT3/4								23 0	
**Trinh et al.** **[27]**	**87**	**A, C**	**2012**	**SALL4** **CD117** **OCT3/4**		**5/31** **9/31** **0/31**	**29/29** **29/29** **0/29**	**4/25** **25/25** **23/25**		**0/2** **2/2** **2/2**			
Cao et al. [28]	31	A	2009	SALL4 OCT3/4 PLAP			4/4 0/4 2/4	7/7 7/7	1/1 1/1		1/1 0/7	0/18	

A = adults, **C = children**, u = unknown data, GCT—germinal cell tumor; ET—epithelial tumors; SCST—sex cord stromal tumor; MT—mature teratoma; IT—immature teratoma; YST—yolk sac tumor; DG—dysgerminoma; EC—embryonal carcinoma; MGC—mixed germ cell tumor; Cc—choriocarcinoma.

## References

[1] Ries L. (1999). Cancer Incidence and Survival among Children and Adolescents: United States SEER Program, 1975–1995.

[2] Hermans A.J., Kluivers K.B., Janssen L.M., Siebers A.G., Wijnen M.H., Bulten J., Massuger L.F., Coppus S.F. (2016). Adnexal masses in children, adolescents and women of reproductive age in the Netherlands: A nationwide population-based cohort study. Gynecol. Oncol..

[3] Cheung A.N., Ellenson L.H., Gilks C.B., Kim K.R., Kong C.S., Lax S.F., Longacre T.A., Malpica A., McCluggage W.G., Oliva E., WHO Classification of Tumours Editorial Board (2020). Tumors of the Ovary. Female Genital Tumours.

[4] Backer D., Madern A., Oosterhuis G.C., Hakvoort-Cammel J.W., Hazebroek F. (2006). Ovarian Germ Cell Tumors in Children: A Clinical Study of 66 Patients. Pediatr. Blood Cancer.

[5] Young R.H. (2005). Sex cord-stromal tumors of the ovary and testis: Their similarities and differences with consideration of selected problems. Mod. Pathol..

[6] Fresneau B., Orbach D., Faure-Conter C., Verité C., Castex M.P., Kalfa N., Martelli H., Patte C. (2015). Sex-Cord Stromal Tumors in Children and Teenagers: Results of the TGM-95 Study. Pediatr. Blood Cancer.

[7] Ray-Coquard I., Morice P., Lorusso D., Prat J., Oaknin A., Pautier P., Colombo N. (2018). Non-epithelial ovarian cancer: ESMO Clinical Practice Guidelines for diagnosis, treatment and follow-up. Ann. Oncol..

[8] Critical Peak Pricing—San Diego Gas & Electric. https://www.sdge.com/businesses/savings-center/energy-management-programs/demand-response/critical-peak-pricing.

[9] Ritchie J., Omahony F., Garden A. (2018). Guideline for the Management of Ovarian Cysts in Children and Adolescents. Br. Soc. Paediatr. Adolesc. Gynaecol..

[10] Sessa C., Schneider D.T., Planchamp F., Baust K., Braicu E.I., Concin N., Godzinski J., McCluggage W.G., Orbach D., Pautier P. (2020). ESGO–SIOPE guidelines for the management of adolescents and young adults with non-epithelial ovarian cancers. Lancet Oncol..

[11] Schneider D.T., Orbach D., Ben-Ami T., Bien E., Bisogno G., Brecht I.B., Cecchetto G., Ferrari A., Godzinski J., Janic D. (2021). Consensus recommendations from the EXPeRT/PARTNER groups for the diagnosis and therapy of sex cord stromal tumors in children and adolescents. Pediatr. Blood Cancer.

[12] Akakpo P.K.M., Derkyi-Kwarteng L.M., Quayson S.E.M., Gyasi R.K.M., Anim J.T.M. (2016). Ovarian Tumors in Children and Adolescents: A 10-Yr Histopathologic Review in Korle-Bu Teaching Hospital, Ghana. Int. J. Gynecol. Pathol..

[13] Atılgan A.O., Özen Ö., Reyhan A.N.H., Ayhan A. (2022). Clinicopathologic Features and the Loss of ARID1A Expression in Ovarian Seromucinous Borderline Tumors and Seromucinous Carcinomas. Int. J. Surg. Pathol..

[14] Yang M., Xie X., Ding Y. (2016). SALL4 is a marker of poor prognosis in serous ovarian carcinoma promoting invasion and metastasis. Oncol. Rep..

[15] Cao D., Humphrey P.A., Allan R.W. (2009). SALL4 is a novel sensitive and specific marker for metastatic germ cell tumors, with particular utility in detection of metastatic yolk sac tumors. Cancer.

[16] Wu P., Luo R., Sun B., Zhao J., Xu Q., Feng S., Chen X., Wang C. (2020). SALL4 is a useful marker for pediatric yolk sac tumors. Pediatr. Surg. Int..

[17] Bai S., Wei S., Ziober A., Yao Y., Bing Z. (2012). SALL4 and SF-1 Are Sensitive and Specific Markers for Distinguishing Granulosa Cell Tumors from Yolk Sac Tumors. Int. J. Surg. Pathol..

[18] Yu L., Jiao Y.-J., Zhou L., Song W.-Q., Wu S.-W., Wang D.-N. (2016). Expressions of OCT4, Notch1 and DLL4 and their clinical im-plications in epithelial ovarian cancer. Nan Fang Yi Ke Da Xue Xue Bao.

[19] Zhang J., Li Y.-L., Zhou C.-Y., Hu Y.-T., Chen H.-Z. (2010). Expression of octamer-4 in serous and mucinous ovarian carcinoma. J. Clin. Pathol..

[20] Cheng L., Thomas A., Roth L.M., Zheng W., Michael H., Karim F.W.A. (2004). OCT4. Am. J. Surg. Pathol..

[21] Cheng L., Zhang S., Talerman A., Roth L.M. (2010). Morphologic, immunohistochemical, and fluorescence in situ hybridization study of ovarian embryonal carcinoma with comparison to solid variant of yolk sac tumor and immature teratoma. Hum. Pathol..

[22] Chang M.C., Vargas S.O., Hornick J.L., Hirsch M.S., Crum C.P., Nucci M.R. (2009). Embryonic Stem Cell Transcription Factors and D2-40 (Podoplanin) as Diagnostic Immunohistochemical Markers in Ovarian Germ Cell Tumors. Int. J. Gynecol. Pathol..

[23] Salonen J., Leminen A., Stenman U.-H., Bützow R., Heikinheimo M., Heikinheimo O. (2008). Tissue AP-2γ and Oct-3/4, and Serum CA 125 as Diagnostic and Prognostic Markers of Malignant Ovarian Germ Cell Tumors. Tumor Biol..

[24] Abiko K., Mandai M., Hamanishi J., Matsumura N., Baba T., Horiuchi A., Mikami Y., Yoshioka S., Wakasa T., Shiozawa T. (2010). Oct4 Expression in Immature Teratoma of the Ovary. Am. J. Surg. Pathol..

[25] Mosbech C.H., Svingen T., Nielsen J.E., Toft B.G., Rechnitzer C., Petersen B.L., Rajpert-De Meyts E., Hoei-Hansen C.E. (2014). Expression pattern of clinically relevant markers in paediatric germ cell- and sex-cord stromal tumours is similar to adult testicular tumours. Virchows Arch..

[26] Miettinen M., Wang Z., McCue P.A., Sarlomo-Rikala M., Rys J., Biernat W., Lasota J., Lee Y.-S. (2014). SALL4 Expression in Germ Cell and Non–Germ Cell Tumors. Am. J. Surg. Pathol..

[27] Trinh D.T., Shibata K., Hirosawa T., Umezu T., Mizuno M., Kajiyama H., Kikkawa F. (2012). Diagnostic utility of CD117, CD133, SALL4, OCT4, TCL1 and glypican-3 in malignant germ cell tumors of the ovary. J. Obstet. Gynaecol. Res..

[28] Cao D., Li J., Guo C.C., Allan R.W., Humphrey P.A. (2009). SALL4 Is a Novel Diagnostic Marker for Testicular Germ Cell Tumors. Am. J. Surg. Pathol..

[29] Talebagha S., Rizk C., Elawabdeh N., Abramowsky C.R., Shehata B.M. (2012). Usefulness of OCT4/3 Immunostain in Pediatric Malignant Germ Cell Tumors. Fetal Pediatr. Pathol..

[30] Karmakar S., Seshacharyulu P., Lakshmanan I., Vaz A.P., Chugh S., Sheinin Y.M., Mahapatra S., Batra S.K., Ponnusamy M.P. (2017). hPaf1/PD2 interacts with OCT3/4 to promote self-renewal of ovarian cancer stem cells. Oncotarget.

[31] Van Nieuwenhuysen E., Busschaert P., Neven P., Han S.N., Moerman P., Liontos M., Papaspirou M., Kupryjanczyk J., Hogdall C., Hogdall E. (2018). The genetic landscape of 87 ovarian germ cell tumors. Gynecol. Oncol..

[32] Yang W.-P., Zou Y., Huang C.-S., Zhang S.-Z., Xiao Q., Dai K.-L., Zhong H.-S., Xiong X.-J. (2007). Clinicopathologic and prognostic study of pediatric immature teratoma. Zhonghua Bing Li Xue Za Zhi.

[33] Wang S., Jia J., Liu D., Wang M., Wang Z., Li X., Wang H., Rui Y., Liu Z., Guo W. (2019). Matrix Metalloproteinase Expressions Play Important role in Prediction of Ovarian Cancer Outcome. Sci. Rep..

[34] Zeng L., Qian J., Zhu F., Wu F., Zhao H., Zhu H. (2019). The prognostic values of matrix metalloproteinases in ovarian cancer. J. Int. Med. Res..

[35] Molenda S., Sikorska A., Florczak A., Lorenc P., Dams-Kozlowska H. (2023). Oligonucleotide-Based Therapeutics for STAT3 Targeting in Cancer—Drug Carriers Matter. Cancers.

[36] Horwitz V., Davidson B., Stern D., Tropé C.G., Re’em T.T., Reich R. (2016). Ezrin Is Associated with Disease Progression in Ovarian Carcinoma. PLoS ONE.

[37] Karthika C., Sureshkumar R., Zehravi M., Akter R., Ali F., Ramproshad S., Mondal B., Tagde P., Ahmed Z., Khan F.S. (2022). Multidrug Resistance of Cancer Cells and the Vital Role of P-Glycoprotein. Life.

[38] Andeen N.K., Tretiakova M.S. (2016). Metastatic Treated Malignant Germ Cell Tumors. Appl. Immunohistochem. Mol. Morphol..

[39] Sun B., Xu L., Bi W., Ou W.-B. (2022). SALL4 Oncogenic Function in Cancers: Mechanisms and Therapeutic Relevance. Int. J. Mol. Sci..

[40] Liu Y., Peng X., Li H., Jiao W., Peng X., Shao J., Xu Y., Wang R., Wang W., Kong D. (2021). STAT3 Inhibitor Napabucasin Inhibits Tumor Growth and Cooperates with Proteasome Inhibition in Human Ovarian Cancer Cells. Recent Pat. Anti-Cancer Drug Discov..

[41] Rizzino A. (2009). Sox2 and Oct-3/4: A versatile pair of master regulators that orchestrate the self-renewal and pluripotency of embryonic stem cells. Wiley Interdiscip. Rev. Syst. Biol. Med..

[42] Hsu L.-J., Liu C.-L., Kuo M.-L., Shen C.-N., Shen C.-R. (2021). An Alternative Cell Therapy for Cancers: Induced Pluripotent Stem Cell (iPSC)-Derived Natural Killer Cells. Biomedicines.

[43] Lin T.-C., Wang K.-H., Chuang K.-H., Kao A.-P., Kuo T.-C. (2023). Oct-4 induces cisplatin resistance and tumor stem cell-like properties in endometrial carcinoma cells. Taiwan J. Obstet. Gynecol..

[44] Abouelnazar F.A., Zhang X., Wang M., Zhang J., Yu D., Zang X., Zhang J., Li Y., Xu J., Yang Q. (2023). The new advance of SALL4 in cancer: Function, regulation, and implication. J. Clin. Lab. Anal..

[45] Schmidtova S., Dorssers L.C.J., Kalavska K., Gillis A.J.M., Oosterhuis J.W., Stoop H., Miklikova S., Kozovska Z., Burikova M., Gercakova K. (2020). Napabucasin overcomes cisplatin resistance in ovarian germ cell tumor-derived cell line by inhibiting cancer stemness. Cancer Cell Int..

